# Beyond therapeutic feeding: a call for integrated strategies to end child malnutrition in Somalia

**DOI:** 10.3389/fpubh.2026.1941811

**Published:** 2026-10-05

**Authors:** Abdifatah Mohamed Tahlil, Mohamed Jama Hashi, Hafsa Mohamed Hassan, Mukhtar Baryare Matan, Abdifatah Hassan Elmi, Ali Mohamud Samatar, Mohamed Abdirahman Abdi, Abdullahi Ahmed Tahlil

**Affiliations:** 1 Pediatric Department, Benadir Maternity and Children Hospital, Mogadishu, Somalia; 2 Faculty of Medicine and Surgery, Mogadishu University, Mogadishu, Somalia; 3 Ministry of Health and Human Services, Federal Government of Somalia, Mogadishu, Somalia; 4 Department of Public Health, Faculty of Graduate Studies and Research, Somali National University, Mogadishu, Somalia; 5 Center for Epidemic Intelligence, Preparedness and Response, Mogadishu, Somalia

**Keywords:** child malnutrition, community-based management of acute malnutrition, food security, humanitarian response, Somalia, stunting, therapeutic feeding, wasting

## Abstract

Somalia records one of the highest global burdens of child wasting and stunting, with approximately 1.7–1.85 million children under five estimated to be affected by acute malnutrition annually during 2022–2025, including approximately 421,000–513,550 children with severe acute malnutrition. For over two decades, the dominant policy response has been community-based management of acute malnutrition (CMAM), a curative intervention that treats clinical endpoints rather than underlying drivers. Despite meeting international cure-rate standards, CMAM alone cannot address the food insecurity, water, sanitation and hygiene (WASH), microbial and parasitic infectious diseases, health-system, and climatic drivers that perpetuate malnutrition. This paper integrates recent Somali survey, programme-evaluation, and administrative data to show that the national burden of acute malnutrition has remained high despite continued treatment scale-up, with stunting falling only 9.7 percentage points over 24 years, relapse after discharge remaining a documented challenge, and nationally representative Somalia-specific estimates of CMAM treatment coverage remaining unavailable. Diagnostic discordance between screening tools leaves an unknown share of wasted children undetected, and persistent sex- and region-based disparities highlight structural gaps. Ending child malnutrition in Somalia requires rebalancing investment from an emergency, treatment-centred paradigm toward integrated, multisectoral strategies combining therapeutic feeding with WASH investment, maternal and infant feeding support, primary health-care strengthening, climate-resilient livelihoods, and shock-responsive social protection. We propose four policy shifts: sustaining CMAM while addressing diagnostic gaps, mandating nutrition-sensitive financing, institutionalizing relapse prevention, and expanding anticipatory financing. These shifts require governance reforms and multi-year donor commitment to address the determinants of child nutrition in Somalia comprehensively.

## Introduction

Somalia remains one of the most nutritionally insecure countries in the world. In the 2025 Global Hunger Index, Somalia ranked last among the 123 countries with sufficient data to compute a score, with a value of 42.6 classified as “alarming” (1). At the height of the 2022 drought, humanitarian agencies estimated that 1.8 million children aged 6–59 months were affected by acute malnutrition, including 513,550 children classified with severe acute malnutrition (SAM). The severity of the crisis was reflected in the increase in severe acute malnutrition prevalence, with the median prevalence rising from 1.5% during the 2021 Gu season (the main rainy season in Somalia) to 3.1% during the 2022 Gu season (2). This was not a one-off spike. Analyses by the Food Security and Nutrition Analysis Unit–Somalia (FSNAU) and the Integrated Food Security Phase Classification (IPC) indicate that the burden of acute malnutrition remained persistently high, with approximately 1.8 million children affected in 2023, including 477,700 children with SAM. In 2024, the estimated burden remained substantial, with approximately 1.85 million children experiencing acute malnutrition, including 475,000 severely wasted children. Projections for 2025 indicate a continued critical situation, with approximately 1.85 million children expected to suffer from acute malnutrition, including 421,000 children with SAM (3, 4).

The burden described above encompasses distinct forms of child malnutrition with different causes, trajectories, and programmatic implications. Wasting is an acute condition characterized by low weight-for-height, reflecting recent nutritional deterioration often associated with illness, inadequate intake, or acute shocks, and is the primary focus of interventions such as community-based management of acute malnutrition (CMAM). In contrast, stunting is a chronic condition characterized by low height-for-age, resulting from prolonged nutritional deprivation, recurrent microbial and parasitic infections, and inadequate caregiving and environmental conditions, particularly during the first 1,000 days of life (5, 6). While therapeutic feeding remains essential for reducing mortality from wasting, it cannot address the broader structural determinants underlying chronic growth failure.

Chronic undernutrition tells a similarly sobering, if slightly more encouraging, story. A secondary analysis of World Health Organization (WHO) model-based estimates found that national stunting prevalence in Somalia fell from 33.6% in 2000 to 23.9% in 2024, a reduction of 9.7 percentage points over 24 years, but that sex-based disparities persisted throughout, with stunting affecting 25.7% of boys and 22.0% of girls in 2024 and an inequality ratio that barely moved across the study period (5). A nationally representative micronutrient survey conducted in 2019, using a different methodology and case definition, found lower stunting (17.2%) and wasting (11.0%) prevalence nationally (6), while the 2020 Somaliland Demographic and Health Survey reported materially higher rates in Somaliland, specifically 23.4% stunting and 21.3% wasting (7). These differences across surveys are partly methodological, but they also illustrate genuine sub-national heterogeneity that a single national treatment target cannot capture. Differences may also reflect variation in sampling frames, geographical coverage, seasonality, measurement procedures, and population characteristics.

The dominant programmatic response to this burden has been the scale-up of community-based management of acute malnutrition (CMAM), built around ready-to-use therapeutic food (RUTF), outpatient treatment for uncomplicated severe acute malnutrition (SAM), and inpatient stabilization for children with medical complications. This model has demonstrably saved lives. Yet the persistence of the burden, together with programme-level evidence on coverage gaps and relapse reviewed below, indicates that treatment scale-up alone has not coincided with a sustained reduction in the underlying national burden. This paper integrates recent Somali surveys, programme evaluations, and administrative data to argue that ending child malnutrition in Somalia requires deliberately rebalancing the response toward the food-security, WASH, health-system, infant-feeding, and climate drivers that determine whether a child becomes malnourished in the first place.

This paper contributes to policy and practice by synthesizing recent (2019–2025) Somali-specific evidence from surveys, programme evaluations, and peer-reviewed studies to provide a consolidated understanding of the limitations of a treatment-centred approach. Rather than generating new empirical findings, this paper translates emerging evidence on diagnostic challenges, relapse, treatment coverage limitations, and underlying determinants into actionable policy recommendations for the Federal Government of Somalia and humanitarian partners.

## Approach and evidence synthesis

This paper synthesizes publicly available data from Somali national surveys (FSNAU/IPC acute malnutrition analyses 2019–2025; Somalia Micronutrient Survey 2019; Somaliland Demographic and Health Survey 2020), WHO model-based estimates (2000–2024), programme evaluations of CMAM in Somalia and the ESA region, and peer-reviewed literature on malnutrition determinants in Somalia identified through PubMed, Scopus, and Google Scholar search terms included combinations of Somalia, child malnutrition, wasting, stunting, CMAM, therapeutic feeding, intestinal parasitic infections, parasitic diseases, and childhood infections. This article is a policy-focused perspective informed by a structured narrative evidence synthesis. We did not undertake a full systematic review; instead, we applied a transparent and reproducible approach to identify, select, and interpret relevant evidence from Somalia-specific and international sources. The synthesis aimed to characterize the burden and determinants of child malnutrition in Somalia, evaluate the strengths and limitations of the current treatment-centred response, and identify evidence-informed opportunities for integrated nutrition strategies. Evidence was organized according to four domains: (1) magnitude and trends of child malnutrition; (2) determinants and drivers of malnutrition; (3) performance and limitations of therapeutic feeding approaches; and (4) multisectoral strategies for prevention and sustainable reduction of malnutrition.

## Evidence identification and selection

Evidence sources included peer-reviewed studies, national surveys, programme evaluations, humanitarian assessments, policy documents, and international technical reports. Searches were conducted in PubMed, Scopus, and Google Scholar between January and March 2026. Additional grey literature was identified through publications from the Federal Ministry of Health and Human Services Somalia, FSNAU/IPC, WHO, UNICEF, FAO, and other humanitarian nutrition partners. Eligible evidence sources included studies and reports addressing children under 5 years of age in Somalia or comparable fragile settings, focusing on malnutrition prevalence, determinants, CMAM performance, relapse, screening approaches, WASH, infant feeding, health-system interventions, food security, livelihoods, and social protection. Sources were excluded if they did not provide relevant nutritional outcomes, lacked methodological descriptions, or were unrelated to child nutrition policy and programming. Two authors independently reviewed identified evidence sources, extracted relevant information, and resolved disagreements through discussion with the wider author team. Because this was a perspective article rather than a systematic review, formal risk-of-bias assessment was not conducted. However, evidence credibility was considered based on study design, methodological quality, relevance to Somalia, and consistency with findings from multiple sources. Somalia-specific empirical evidence was prioritized when available, while international evidence was used to inform interpretation of intervention mechanisms and policy implications.

### Analytical framework

The synthesis was guided by a conceptual framework linking the immediate, underlying, and structural determinants of child malnutrition in Somalia. The framework considers child malnutrition as the result of interactions between nutritional status, disease burden, household food insecurity, environmental conditions, health-system capacity, climate shocks, and governance factors. This analytical approach is consistent with the UNICEF conceptual framework of malnutrition, which recognizes that child nutritional outcomes result from the interaction of immediate causes, including inadequate dietary intake and disease; underlying causes, including household food insecurity, inadequate caregiving practices, insufficient health services, and poor water, sanitation, and hygiene conditions; and broader structural determinants related to socioeconomic conditions, governance, and political and environmental factors. Applying this framework allows the present synthesis to move beyond clinical treatment outcomes and examine the interconnected systems that shape child nutrition in Somalia (5, 6).

The framework consisted of four interconnected components:

1. Burden and distribution of malnutrition: Assessment of wasting, severe wasting, and stunting trends among Somali children.
2. Determinants and drivers: Examination of food insecurity, microbial and parasitic infectious diseases, WASH conditions, infant feeding practices, health-system limitations, climate variability, and displacement.
3. Current response assessment: Evaluation of CMAM and therapeutic feeding performance, including coverage limitations, diagnostic challenges, and relapse.
4. Integrated policy response: Identification of multisectoral strategies linking nutrition-specific interventions with WASH, primary healthcare, social protection, livelihoods, and climate adaptation.

This conceptual pathway is illustrated in Figure 1, which summarizes the relationship between the determinants of child malnutrition, limitations of current treatment-centred approaches, and integrated policy solutions.

**Figure 1:**
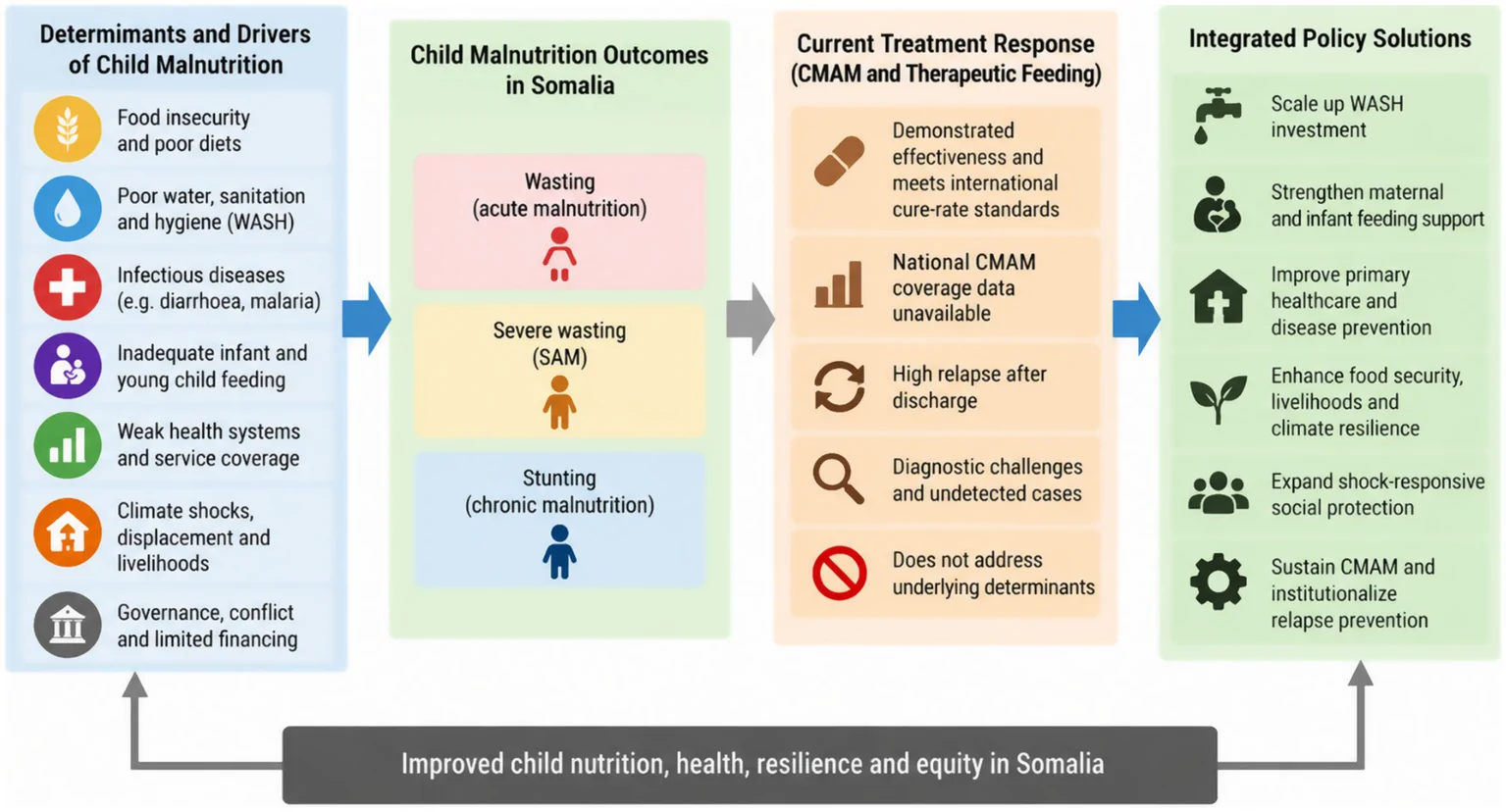
Conceptual framework linking determinants of child malnutrition, current treatment response gaps, and integrated policy solutions in Somalia.

## The scale and persistence of child malnutrition in Somalia

Child malnutrition in Somalia is best understood as the product of overlapping and mutually reinforcing shocks rather than a single humanitarian event. The 2020–2023 period saw five, and in some areas six, consecutive failed rainy seasons, the longest sequence on record, which displaced hundreds of thousands of people, killed an estimated 3.5 million livestock, and eroded the pastoral and agro-pastoral livelihoods on which most rural Somali households depend (8, 9). A mortality study commissioned by UNICEF and the World Health Organization and conducted by the London School of Hygiene and Tropical Medicine and Imperial College London estimated approximately 43,000 excess deaths in Somalia in 2022 alone, with children under five accounting for roughly half of that toll (10). The International Monetary Fund’s 2022 country assessment identified Somalia’s narrow arable land base, just 1.8% of total land area, high dependence on rain-fed and pastoral production, recurrent locust infestation, and structurally weak food systems as long-standing drivers of food insecurity that predate and will outlast any single drought cycle (11). By 2025, renewed drought conditions, protracted conflict, and elevated food prices were again projected to affect approximately 1.85 million children with acute malnutrition (Figure 2), underscoring that Somalia’s nutrition crisis is cyclical and structurally embedded rather than exceptional (4).

**Figure 2:**
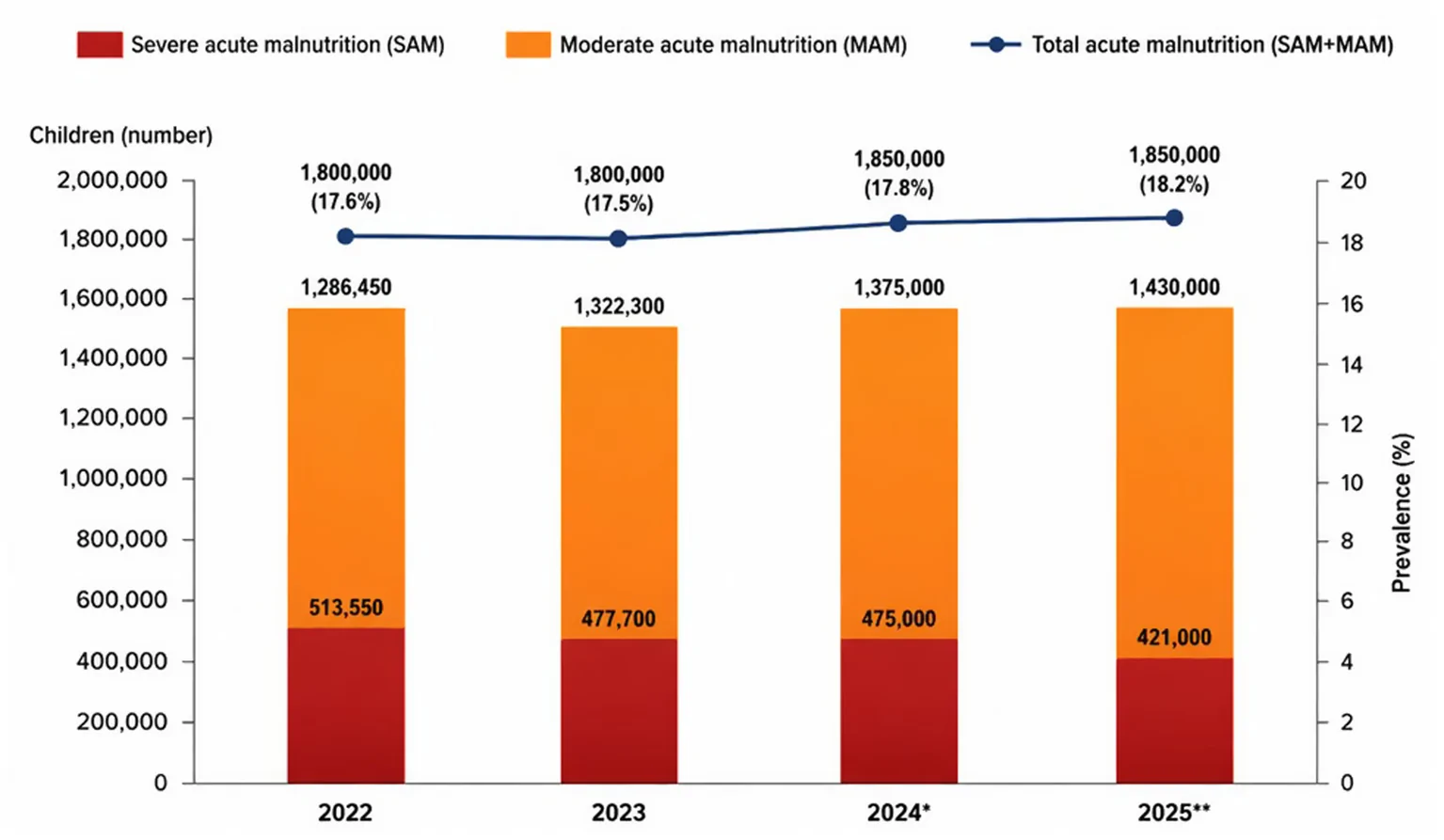
Burden of acute malnutrition among Somali children under five, 2022–2025.

Malnutrition in Somalia is also geographically and demographically uneven. Analyses restricted to Mogadishu found that child age, birth order, short birth intervals, parental education, and maternal occupation were all independently associated with the likelihood of protein-energy malnutrition, indicating that household-level social and economic conditions interact with the macro-level drivers of drought and displacement (12). Regional disparities are similarly stark: the Banadir region encompassing Mogadishu has at times been projected to have the highest proportion of acutely malnourished children of any region in the country, even though it is comparatively more urbanized and closer to health infrastructure than rural pastoral areas (13), while the sex-based stunting gap documented nationally has remained essentially unchanged for 24 years (5), suggesting that neither urban proximity to services nor two decades of programming have been sufficient to close these structural gaps.

## The predominant paradigm: therapeutic and curative feeding

CMAM has been the backbone of the nutrition response in Somalia and across eastern and southern Africa (ESA) for close to two decades. A recent multi-country analysis of CMAM performance in sixteen ESA countries found that Ethiopia, Somalia, and South Sudan together accounted for 72.6% of all CMAM admissions recorded in the region in 2021, and that programmes meeting Sphere minimum standards for cure and mortality rates averted an estimated 150,000 child deaths that year alone, treating 1,164,196 children with severe wasting overall, equivalent to only 33.9% of the estimated regional caseload (14). A systematic review and meta-analysis focused specifically on integrated management of acute malnutrition programmes in Somalia similarly concluded that treatment produces high pooled recovery rates among children who complete the programme (15). Even under the operational disruption of the coronavirus disease 2019 (COVID-19) pandemic, adapted CMAM protocols in Uganda, Ethiopia, and Somalia continued to deliver stable admission, length-of-stay, and recovery outcomes, demonstrating the resilience of the delivery model under stress (16).

These achievements are real and should not be understated. Ready-to-use therapeutic food (RUTF)-based outpatient treatment and community-based management of acute malnutrition (CMAM) are among the most extensively validated interventions for reducing mortality among children with severe wasting. Global guidance from the World Health Organization recommends these interventions as essential components of acute malnutrition management while emphasizing their integration within broader health, nutrition, and community systems rather than their implementation as isolated emergency responses (14, 15). However, evidence from multiple settings indicates that treatment effectiveness alone cannot eliminate malnutrition when underlying determinants such as food insecurity, infection exposure, poor WASH conditions, and limited healthcare access remain unresolved. The argument advanced here is not that therapeutic feeding should be curtailed, but that it has, in practice, become the default and often singular organizing framework for the nutrition response, with prevention, WASH, health-system strengthening, and livelihoods programming persistently under-resourced by comparison. The regional coverage estimate cited above should therefore not be interpreted as a Somalia-specific treatment-coverage estimate. Nevertheless, the absence of nationally representative CMAM coverage data for Somalia highlights an important evidence gap in assessing whether therapeutic services are reaching children who need them.

## Limitations of a treatment-centred approach

### Relapse and post-discharge vulnerability

A growing body of evidence indicates that recovery from severe acute malnutrition is frequently not sustained. A prospective cohort study following children discharged from CMAM programmes in Somalia’s Bay and Hiran regions found that a substantial proportion relapsed into acute malnutrition within months of recovery, and identified household food insecurity, water access, and caregiving conditions as key predictors of relapse (17). A companion multi-country economic analysis conducted in Mali, Somalia, and South Sudan quantified the direct cost implications of this pattern, showing that treating a relapsed case adds materially to the cost of an initial CMAM episode and strains already constrained humanitarian budgets (18, 19). Repeated relapse suggests that treatment alone may not sufficiently address the underlying conditions affecting recovery, including household food insecurity, water access, infection exposure, and caregiving conditions.

### Measurement and coverage gaps

Somalia has also been identified as a setting with substantial discordance between commonly used measures of acute malnutrition. Comparative analyses of mid-upper arm circumference (MUAC), MUAC-for-age z-score, and weight-for-height z-score (WHZ) in Somali children have found an 8–10 percentage-point discordance between the wasting prevalence estimated by MUAC and that estimated by WHZ, with MUAC-based screening tending to underestimate the true burden (20). Because MUAC is the primary tool used for community screening and CMAM admission in Somalia, this discordance may result in some children with wasting being missed by routine screening. However, the extent of this potential under-detection at national level remains uncertain. The 33.9% coverage estimate reported above refers to the combined caseload across 16 Eastern and Southern African countries and should not be interpreted as a Somalia-specific coverage estimate (14). Nationally representative estimates of CMAM treatment coverage for Somalia remain unavailable, limiting direct assessment of programme reach at country level.

### Stunting: the burden therapeutic feeding cannot reach

CMAM is designed to treat acute wasting; it is not designed to prevent or reverse stunting, the chronic linear growth faltering associated with irreversible cognitive and economic costs across the life course. National stunting prevalence fell by 9.7 percentage points between 2000 and 2024, during a period in which emergency nutrition programmes were also scaled up (5). Because stunting is influenced by multiple interacting determinants, this temporal association does not permit attribution of the observed trend to therapeutic feeding programmes. The 2019 Somalia Micronutrient Survey found stunting to be strongly associated with household food insecurity (adjusted risk ratio [aRR] 1.47), systemic inflammation (aRR 1.75), and iron deficiency (aRR 2.09) (6), factors rooted in chronic dietary inadequacy and infection rather than acute shock. Because stunting accumulates over the first 1,000 days of life and is driven by cumulative nutrient and infection exposure rather than a single acute episode, it cannot be meaningfully addressed by an emergency feeding programme that is, by design, activated only after a child has already become acutely wasted. This understanding is consistent with broader evidence that sustainable reductions in child undernutrition require coordinated investments across nutrition-specific interventions, maternal and child health services, food systems, infection prevention, and enabling social and economic environments. The evidence highlights that improvements in child nutrition depend not only on treatment of clinical manifestations but also on addressing the broader determinants affecting maternal nutrition, infant feeding, household resources, and access to essential services (12, 16).

## Toward integrated strategies

Somalia’s own policy architecture already recognizes the need for a broader approach. The Federal Government’s Multisectoral Nutrition Strategy explicitly adopts the Scaling Up Nutrition (SUN) movement’s distinction between nutrition-specific interventions, such as CMAM, micronutrient supplementation, and infection management, and nutrition-sensitive interventions that address the underlying and basic causes of malnutrition through food access, water and sanitation, health care, and social protection (21). The gap in Somalia has not primarily been one of strategic design but of implementation: nutrition-sensitive sectors remain chronically under-financed relative to emergency feeding, and coordination across the agriculture, water, health, and social-protection ministries responsible for delivering them remains weak. The need for integrated action is also consistent with global nutrition evidence demonstrating that no single intervention can independently eliminate child malnutrition. Successful nutrition improvements require coordinated action across healthcare delivery, food security, WASH services, infant and young child feeding support, social protection, and resilient livelihoods (15). This evidence reinforces the importance of aligning Somalia’s emergency nutrition response with long-term systems strengthening and prevention-oriented investments.

### Water, sanitation, and hygiene (WASH)

The relationship between WASH conditions and child nutritional status is biologically plausible and increasingly documented in Somali data. A hierarchical, multilevel analysis of the 2020 Somaliland Demographic Health Survey found that improved sanitation was associated with significantly lower odds of stunting (adjusted odds ratio [AOR] 0.69, 95% CI 0.56–0.86), while unimproved sanitation increased the odds of wasting (AOR 1.30, 95% CI 1.02–1.68); children in households requiring more time to collect drinking water also had higher odds of stunting (AOR 1.39, 95% CI 1.11–1.75) even after adjusting for household and community-level confounders (7). A recent review of Somalia’s public health challenges similarly identified inadequate WASH infrastructure as one of the five major public-health challenges identified in the review of the country’s combined burden of malnutrition, infectious disease, and child mortality (22). Evidence from international studies indicates that repeated exposure to faecal pathogens during early childhood may contribute to environmental enteric dysfunction, a subclinical intestinal disorder associated with impaired intestinal barrier function and nutrient absorption. However, the magnitude of this pathway and its contribution to child malnutrition vary according to environmental, socioeconomic, and implementation contexts.

Embedding water-point rehabilitation, latrine construction, and hygiene promotion within nutrition programming represents an important component of a multisectoral nutrition strategy in Somalia. However, most Somalia-specific evidence linking WASH conditions with nutritional outcomes is observational and cannot establish causality. Furthermore, evidence from global WASH-nutrition intervention trials suggests that impacts depend on intervention intensity, population coverage, implementation quality, and the wider social and economic context. Nevertheless, given Somalia’s high burden of infectious diseases and inadequate WASH infrastructure, integrating WASH interventions with nutrition services represents a potentially important strategy for addressing multiple pathways contributing to child malnutrition.

### Maternal, infant, and young child feeding

Exclusive breastfeeding for the first 6 months of life, followed by the timely introduction of nutritionally adequate complementary foods with continued breastfeeding up to 2 years and beyond, represents one of the most effective and affordable strategies for improving child survival, growth, and development. Studies emphasize that optimal infant and young child feeding practices are central components of preventing child malnutrition and should be integrated into routine maternal, newborn, and child health platforms (23, 24). However, Somali evidence indicates persistent barriers related to maternal education, social support, socioeconomic conditions, and limited access to counselling services. The 2019 national survey found that the association between recent diarrhea and wasting was substantially attenuated among exclusively breastfed infants aged 0–5 months (6). A scoping review of exclusive breastfeeding among Somali children found that higher rates were associated with maternal education, antenatal-care attendance, and social support, while low socioeconomic status, cultural beliefs about milk sufficiency, and limited health-facility contact were recurrent barriers (23). District- and facility-level studies in Banadir and Puntland similarly report incomplete exclusive breastfeeding linked to inadequate counselling during antenatal and postnatal contacts and to competing caregiving demands among displaced and low-income mothers (24, 25). Because infant and young child feeding counselling can be delivered through the same maternal and child health platforms that already provide antenatal care, immunization, and growth monitoring, strengthening this counselling represents a comparatively low-cost, high-yield addition to the existing health-system footprint rather than a parallel programme requiring new infrastructure.

### Primary health-system strengthening

Malnutrition and infectious disease act synergistically, a relationship visible directly, where recent diarrhea was the single strongest correlate of wasting identified in the 2019 national survey (aRR 3.51) (6). A review of Somalia’s public health landscape identified fragile primary-care infrastructure, disease-outbreak vulnerability, and high maternal and child mortality as interlinked challenges requiring coordinated rather than siloed responses, and explicitly called for multisectoral collaboration and sustained investment in local health-system capacity as preconditions for durable improvement (22). Integrating routine growth monitoring, immunization, oral rehydration and zinc provision for diarrhea, and management of common childhood infections into the same primary-care contact points used for nutrition screening would reduce the disease burden that both precipitates acute malnutrition and undermines recovery after treatment.

Beyond diarrheal illnesses, parasitic infections represent an important but insufficiently recognized contributor to child malnutrition in Somalia. A recent study conducted among children with acute malnutrition in Bay and Banadir regions found a high prevalence of intestinal parasitic infections (82.9%), with *Ascaris lumbricoides*, *Giardia lamblia*, and *Entamoeba histolytica* among the most commonly identified parasites (26). These infections may contribute to poor nutritional outcomes through mechanisms including impaired nutrient absorption, chronic inflammation, appetite reduction, and micronutrient depletion. In the Somalia context, these findings reinforce the potential value of integrating infection prevention, sanitation improvement, and appropriate deworming within nutrition programmes (26).

### Climate-resilient livelihoods and food systems

Because drought is now a recurrent rather than exceptional feature of the Somali climate, nutrition programming cannot be sequenced to follow crisis declarations; it must be embedded in longer-term livelihood and climate-adaptation planning. Reviews of the 2021–2023 drought emphasize that livestock loss (an estimated 3.5 million animals), the collapse of pastoral livelihoods, and the destruction of rain-fed cropland were as consequential for child nutrition outcomes as the rainfall deficit itself (8, 9), implying that drought-resilient livestock and crop interventions, water-harvesting infrastructure, and diversified income sources for pastoral and agro-pastoral households are nutrition interventions in function even when they are not labelled as such. Anticipatory action, releasing pre-positioned resources for cash transfers, livestock support, and supplementary feeding ahead of a forecast poor rainy season rather than after malnutrition indicators have already deteriorated, offers a mechanism to break the cycle evident in Figure 2, in which the acute malnutrition burden remained approximately 1.7–1.85 million children during 2022–2025 despite continued treatment efforts. For example, integrating water-harvesting infrastructure with nutrition programming in drought-prone districts could serve as a concrete model where WASH and livelihoods interventions are co-located with CMAM sites to address both immediate and underlying causes simultaneously.

### Shock-responsive social protection and multisectoral governance

Finally, the evidence reviewed above points toward a governance rather than a purely technical solution: Somalia’s own Multisectoral Nutrition Strategy already articulates a multisectoral framework, and economic analyses of relapse indicate that repeated treatment carries additional costs, providing an economic rationale for considering investments in post-discharge support and prevention (18, 21). What is required is that donor financing should be complemented by greater and more predictable investment in nutrition-sensitive sectors, including WASH, primary care, cash-based social protection, and livelihoods, on a multi-year rather than crisis-triggered basis, and that a single coordinating authority within the Federal Government be resourced to hold food-security, health, water, and social-protection actors accountable to shared nutrition outcome targets rather than sector-specific output indicators.

The gap between Somalia’s Multisectoral Nutrition Strategy and its implementation reflects broader governance challenges, including fragmented donor financing, weak ministry coordination mechanisms, and limited capacity for joint planning and budgeting across health, agriculture, water, and social protection sectors. Addressing these governance barriers is as critical as the technical design of interventions.

## Policy recommendations

Based on the evidence synthesized, we propose four actionable shifts for Somali and donor nutrition policy.

### Sustain CMAM while improving diagnostic accuracy and equitable access

The Federal Ministry of Health (FMOH), together with implementing partners, should maintain CMAM as a core life-saving intervention while strengthening identification and referral pathways. As an initial implementation milestone, high-risk districts could progressively adopt integrated screening approaches combining mid-upper arm circumference (MUAC) and weight-for-height assessment where feasible, subject to operational assessment, training, and resource availability. Priority should be given to conflict-affected and remote communities through mobile nutrition teams and community health worker platforms.

### Establish nutrition-sensitive financing mechanisms

The FMOH should lead the development of a joint nutrition financing framework with the Ministries responsible for Water, Agriculture, Livestock, and Social Affairs. Rather than allocating resources predominantly toward emergency treatment commodities, financing should progressively support prevention-oriented interventions including WASH services, maternal and child health, infant feeding counselling, and social protection. As an illustrative policy option, nutrition-sensitive financing could be considered for progressive expansion toward 30% by 2030. This figure is not an established global requirement or evidence-based universal benchmark; it should be treated as a proposal for policy discussion and refined through national costing, government budgeting processes, prioritization, and stakeholder consultation, while maintaining adequate resources for life-saving therapeutic services.

### Institutionalize post-discharge follow-up and relapse prevention

The FMOH Nutrition Directorate should establish a national post-CMAM follow-up framework integrated within primary healthcare services. The minimum package should include household assessment, growth monitoring, breastfeeding and complementary feeding counselling, referral for food-security support, and linkage with cash-transfer or livelihood programmes where available. Implementation could be initiated in high-burden districts over an initial 12–24-month period, subject to feasibility assessment, resource availability, and integration with existing community health worker structures. Financing should be integrated into nutrition programme budgets rather than relying exclusively on short-term emergency funding.

### Expand anticipatory and shock-responsive financing

The Government of Somalia and development partners should strengthen forecast-based financing mechanisms linked to early warning systems, including FSNAU and IPC monitoring. Resources for cash assistance, livestock protection, water interventions, and nutrition services should be released before drought conditions translate into severe nutritional deterioration.

Implementation of integrated nutrition strategies in Somalia must account for the operational realities of protracted conflict and insecurity. In many areas, insecurity restricts humanitarian access, limits routine health-service delivery, disrupts supply chains, and weakens government coordination capacity. Therefore, integrated approaches should adopt flexible delivery models, including mobile health and nutrition teams, community-based platforms, decentralized supply mechanisms, and stronger engagement with local authorities and community structures. Conflict-sensitive programming is essential to ensure that vulnerable populations in inaccessible and displacement-affected areas are not excluded from prevention and treatment services (Table 1).

**Table 1:** Implementation roadmap for integrated nutrition strategies in Somalia.

Recommendation	Lead actors	Immediate actions	Key constraints	Monitoring indicators
Sustain CMAM and improve diagnosis	MoHHS, partners	Integrate MUAC and WHZ screening	Access, workforce	Screening coverage
Nutrition-sensitive financing	MoHHS, Ministries of Agriculture/WASH	Develop financing framework	Fragmented funding	% prevention financing
Relapse prevention	Nutrition Directorate	Post-discharge follow-up	Funding sustainability	Relapse rate
Anticipatory financing	Government, donors	Link financing with early warning	Coordination	Response time

## Limitations

This synthesis has several limitations. First, although evidence identification followed a structured approach, it was not conducted as a full systematic review and therefore may be subject to selection bias or incomplete retrieval of relevant evidence. Second, included evidence sources varied in methodology, geographic coverage, population characteristics, case definitions, and time periods, limiting direct comparison across studies. Third, much of the Somalia-specific evidence is observational; therefore, causal interpretation of associations between determinants and nutritional outcomes should be made cautiously. Fourth, nationally representative data remain limited for some indicators, including CMAM treatment coverage, and available estimates may not fully capture substantial regional and population-level variation. Finally, evidence regarding the effectiveness and operational feasibility of integrated nutrition interventions in Somalia remains limited. Implementation of proposed strategies will depend on local security conditions, governance capacity, humanitarian access, and sustainable financing mechanisms.

## Conclusion

Therapeutic feeding has saved the lives of many Somali children and remains an indispensable component of the nutrition response. However, the evidence synthesized in this perspective indicates that treatment alone cannot address the multiple determinants that contribute to child malnutrition, including recurrent drought, inadequate water and sanitation systems, recurrent microbial and parasitic infections, suboptimal infant feeding practices, limited primary healthcare capacity, food insecurity, and structurally weak livelihoods. The national acute malnutrition burden remained approximately 1.7–1.85 million children during 2022–2025, while nationally representative Somalia-specific estimates of CMAM treatment coverage remain unavailable. Evidence of post-discharge relapse and persistent chronic undernutrition further highlights the importance of complementing treatment with preventive and systems-level interventions. An integrated approach combining CMAM with WASH, infection prevention and control, maternal and infant feeding support, primary healthcare strengthening, resilient livelihoods, and shock-responsive social protection may provide a more comprehensive framework for addressing both immediate nutritional needs and the underlying conditions that contribute to malnutrition. Progress will require sustained, multisectoral coordination, context-appropriate financing, and continued evaluation of implementation feasibility and effectiveness in Somalia.

## Data Availability

The datasets presented in this study can be found in online repositories. All data analyzed are from publicly available sources cited in the reference list, including FSNAU/IPC reports, WHO estimates, the Somalia Micronutrient Survey, and the Somali Demographic and Health Survey. No new datasets were generated for this paper.

## References

[1] Global Hunger Index. Somalia. Bonn Dublin: Welthungerhilfe and Concern Worldwide (2025).

[2] World Health Organization Regional Office for the Eastern Mediterranean. Somalia Calls for Help as 1.8 Million Somali Children under 5 Experience Acute Malnutrition and Health Complications. Cairo: WHO EMRO (2023).

[3] Food Security and Nutrition Analysis Unit–Somalia, Integrated Food Security Phase Classification. Somalia: Acute Food Insecurity and Malnutrition Snapshot, January–June 2024. Nairobi: IPC/FSNAU (2024).

[4] Food and Agriculture Organization of the United Nations. Somalia: Drought, Conflict and High Food Prices Risk Pushing 4.4 Million People into Hunger, the Federal Government of Somalia and UN Warn. Rome: FAO (2025).

[5] Ali AS Osborne A. Trends in stunting prevalence and sex-based disparities among children under five in Somalia, 2000–2024: a secondary analysis of WHO model-based estimates. J Epidemiol Glob Health. (2026) 16:63. doi: 10.1007/s44197-026-00579-y, 42184021 PMC13212847

[6] Donkor WES Mbai J Sesay F Ali SI Woodruff BA Hussein SM . Risk factors of stunting and wasting in Somali pre-school age children: results from the 2019 Somalia micronutrient survey. BMC Public Health. (2022) 22:264. doi: 10.1186/s12889-021-12439-4, 35139826 PMC8827289

[7] Dahir HM Korse AH Mohamed FI. Water, sanitation, and hygiene and their association with child undernutrition: a hierarchical analysis of the 2020 Somaliland demographic health survey. Front Public Health. (2026) 13:1734101. doi: 10.3389/fpubh.2025.1734101, 41613060 PMC12849758

[8] The impact of drought on food security in Somalia: a comprehensive review. Open Access J (2024). Available online at: https://www.opastpublishers.com/open-access-articles/the-impact-of-drought-on-food-security-in-somalia-a-comprehensive-review-8512.html (Accessed July 15, 2026).

[9] Eklöw K Krampe F. Climate, Peace and Security Fact Sheet: Somalia. Oslo: Norwegian Institute of International Affairs (NUPI) (2023).

[10] United Nations Children's Fund. New Study Finds That 43,000 “Excess Deaths” May Have Occurred in 2022 from the Drought in Somalia. New York: UNICEF (2023).

[11] International Monetary Fund. Somalia: selected issues. IMF Staff Country Rep. (2022) 2022. doi: 10.5089/9798400227899.002

[12] Martin-Canavate R Custodio E Yusuf A Molla D Fasbender D Kayitakire F. Malnutrition and morbidity trends in Somalia between 2007 and 2016: results from 291 cross-sectional surveys. BMJ Open. (2020) 10:e033148. doi: 10.1136/bmjopen-2019-033148, 32071180 PMC7045078

[13] Save the Children International. Somalia: Two in Five Children to Suffer from Acute Malnutrition Despite Rains. Mogadishu: Save the Children Internation (2023).

[14] Baye K Hussein A Angood C Tamene A Woldegiorgis TD Achen JH . Effectiveness of the community management of acute malnutrition in 16 eastern and southern African countries. Nutrition. (2025) 139:112852. doi: 10.1016/j.nut.2025.112852, 40592000

[15] Renzaho AMN Jaiswal C Chineka A Aden MO Dahir A Kari HA . Evaluating the performance of integrated management of acute malnutrition programs in Somalia: a systematic review and meta-analysis. Int J Environ Res Public Health. (2025) 22:378. doi: 10.3390/ijerph22030378 PMC11942193 40238406

[16] Shragai T Talley L Summers A Behringer H Wrabel M Stobaugh H . Outcomes after acute malnutrition program adaptations to COVID-19, Uganda, Ethiopia, and Somalia. Emerg Infect Dis. (2022) 28:S288–98. doi: 10.3201/eid2813.212266, 36502417 PMC9745232

[17] Alier KK Walton S Grounds S Garretson S Mohamoud SA Nur MA . Levels and determinants of child wasting relapse: a prospective cohort study from Somalia. J Glob Health. (2026) 16:04019. doi: 10.7189/jogh-16-04019 PMC12922464 41717987

[18] Puett C King S Chabi SM Mohamud FA Lamwaka N Stobaugh H. The economic implications of relapse among children recovered from severe acute malnutrition: results from a multicountry prospective study in Mali, Somalia, and South Sudan. Am J Clin Nutr. (2026). 10, 107616. doi: 10.1016/j.ajcnut.2026.01.015 PMC12808978 41551722

[19] Puett C King S Stobaugh H. A multi-country, prospective cohort study to evaluate the economic implications of relapse among children recovered from severe acute malnutrition: a study protocol. BMC Nutr. (2022) 8:139. doi: 10.1186/s40795-022-00630-0 PMC9701364 36435873

[20] Garretson S Walton S Alier KK Grounds S Mohamoud SA Nur MA . Analysing concordance between MUAC, MUACZ, and WHZ in diagnosing acute malnutrition among children under five in Somalia. J Glob Health. (2025) 15:04258. doi: 10.7189/jogh.15.04258 PMC12699285 41383165

[21] Federal Republic of Somalia, Ministry of Health and Human Services. Somalia Multisectoral Nutrition Strategy 2019–2024. Mogadishu: Federal Government of Somalia (2019).

[22] Abdi YH Abdi MS Bashir SG Ahmed NI Abdullahi YB. The state of public health in Somalia: top 5 challenges and strategies for improvement. Public Health Chall. (2025) 4:e70138. doi: 10.1002/puh2.70138, 41080794 PMC12509034

[23] Hassan MS Ahmed SA Mohamed FA Abdillahi MA. Exclusive breastfeeding practices among Somali children: a scoping review. Discov Public Health. (2025) 22:609. doi: 10.1186/s12982-025-01017-3

[24] Abdirahman MA Mogere D Musotsi AA Omoto AL. Assessment of prevalence and determinants of exclusive breastfeeding among mothers with infants aged under six months in Wadajir District, Banadir region, Somalia. Int J Trop Dis Health. (2024) 45:12–28. doi: 10.9734/ijtdh/2024/v45i51530

[25] Mahamed AA Mahamed AJ Mohamed AI Osman M Al-Mamun M Muhammad F. Exploring exclusive breastfeeding practices among infants: a comprehensive analysis at Gambool maternal and child health Facility in Garowe, Puntland, Somalia. Cent Afr J Public Health. (2025) 11:87–95. doi: 10.11648/j.cajph.20251103.15

[26] Osman MM Hussein SA Omar MA Issack MM Hanafi AMH Ahmed MY . Pattern of intestinal parasitic infections in children with malnutrition in Somalia. IJID Reg. (2024) 13:100431. doi: 10.1016/j.ijregi.2024.100431, 39391270 PMC11466610

